# The Neurotransmission Basis of Post-Traumatic Stress Disorders by the Fear Conditioning Paradigm

**DOI:** 10.3390/ijms242216327

**Published:** 2023-11-15

**Authors:** Giovanna Traina, Jack A. Tuszynski

**Affiliations:** 1 Department of Pharmaceutical Sciences, University of Perugia, Via Romana, 06126 Perugia, Italy; 2 Department of Mechanical and Aerospace Engineering (DIMEAS), Politecnico di Torino, Corso Duca Degli Abruzzi 24, 10129 Turin, Italy; jackt@ualberta.ca; 3 Department of Data Science and Engineering, The Silesian University of Technology, 44-100 Gliwice, Poland; 4 Department of Physics, University of Alberta, 11335 Saskatchewan Dr NW, Edmonton, AB T6G 2M9, Canada

**Keywords:** contextual fear conditioning, neurotransmission, post-traumatic stress disorders, learning, hippocampus, amygdala, prefrontal cortex

## Abstract

Fear conditioning constitutes the best and most reproducible paradigm to study the neurobiological mechanisms underlying emotions. On the other hand, studies on the synaptic plasticity phenomena underlying fear conditioning present neural circuits enforcing this learning pattern related to post-traumatic stress disorder (PTSD). Notably, in both humans and the rodent model, fear conditioning and context rely on dependent neurocircuitry in the amygdala and prefrontal cortex, cingulate gyrus, and hippocampus. In this review, an overview of the role that classical neurotransmitters play in the contextual conditioning model of fear, and therefore in PTSD, was reported.

## 1. Introduction

Traumatic situations result in a wide range of physiological and neurobiological effects, as well as cognitive distortions and maladaptive behaviors, which include mood disorders, anxiety disorders, personality disorders, and increased risks of incurring a depression state [1].

Post-traumatic stress disorder (PTSD) is a chronic and highly debilitating psychiatric and multifactorial condition that occurs in approximately 6–8% of the general population following exposure to traumatic life events. PTSD represents a significant health and social burden due to its severity and chronicity as well as comorbidity with conditions including major depression [2]. From these observations, it is clear that having cognitive and behavioral experimental models capable of reproducing post-traumatic stress disorder is of great significance. A behavioral model emerged based on a combination of classical conditioning and operant conditioning processes (the two-factor theory proposed by Mowrer in 1947 [3] and the dual conditioning model by Keane et al. [4]).

The brain mechanisms underlying fear are known since it constitutes a vital emotion. On the other hand, an excessively generalized fear is the basis of anxiety disorders; an example of this is provided by PTSD. The fear memory is divided into various stages, such as fear acquisition, fear consolidation, fear reconsolidation, and fear extinction [5]. Consolidation and reconsolidation are transitory phenomena of memory stabilization. During consolidation, the memories are stabilized; while in reconsolidation, the memories are re-stabilized by allowing memory traces to be modified [6]. According to classical conditioning, when exposed to a traumatic event (unconditioned stimulus, US), the subject reacts with a state of fear and a state of high excitement (unconditioned response). Later, he/she continues to show the same response (conditioned response) even when exposed to emotionally neutral stimuli that are associated with the traumatic event (conditioned stimuli, CS). Generally, the conditioned anxious state is extinguished over time, in particular, when the subject is exposed to the conditioned stimulus in the absence of danger [5]. This happens when he/she learns a new association, namely that the conditioned stimulus does not actually signal a dangerous situation. However, the normal extinction of conditioned fear can be hindered by an operant conditioning process, as occurs in PTSD. Therefore, classical (or Pavlovian) fear conditioning is the optimal behavioral paradigm for studying the mechanisms of fear-associative learning and memory processes. And associative learning represents the starting point for any discussion to understand the physiological mechanisms underlying PTSD, in which the components of learning and memory are present. Fear conditioning has been an effective mechanistic pattern related to PTSD and anxiety disorders by etiology [4,7,8]. Conditioning offers a number of crucial advantages for this type of research. One major benefit is that many key elements of conditioned learning are evolutionarily conserved in all animal species, from fruit flies to mammals [9,10]. These experimental paradigms induce rapid learning that persists in the form of long-term memory and provides objective behavioral correlation. Therefore, conditioning constitutes the model of choice for the investigation of PTSD, a study model that is also highly replicated and validated in psychological research.

## 2. Post-Traumatic Stress Disorders (PTSD)

The neurobiology of PTSD is multifactorial and involves endocrine, nervous, genetic, and epigenetic factors. Many studies have shown that the neural changes seen in individuals with PTSD and the brain regions involved are analogous to nerve circuits and fear conditioning areas [11]. The cerebral areas involved are the amygdala, prefrontal cortex, cingulate gyrus, and hippocampus. In particular, the amygdala is principally responsible for processing and regulating “negative” emotions, such as fear and anger. In fact, lesions on the amygdala limit the ability to learn and express fearful conditions [12]. The hippocampus processes short-term memories as well as long-term memories, and it is also important in the recovery of memories related to fear [13].

Studies have also reported that PTSD is accompanied by a reduction in the size of the hippocampus [14]. However, this evidence needs to be fully confirmed.

Other studies have used fear-provoking tasks to activate the system in patients with PTSD. For example, a reduction in the activation of regions of the medial prefrontal cortex has been observed [15]. This evidence is in agreement with the notion of impairment of regulatory processes that promote extinction (which will be referred to in the next section). There is also various evidence of dysfunctions in threat detection, functioning, emotion regulation, and contextual processing [16,17]. Moreover, various studies have shown a reduction in the volume of the PFC as well as a reduction in the connections of this region with subcortical structures depending on trauma [18].

The anterior cingulate cortex is smaller in size and its activity decreases as it is structurally reduced in PTSD conditions, while the anterior midcingulate cortex is reduced but functionally hyperactive [19].

## 3. Contextual Fear Conditioning (CFC)

Fear conditioning constitutes the experimental paradigm that allows researchers to study the neurobiological bases of emotions, and therefore enables them to approach the mechanisms of PTSD [20]. In the contextual fear conditioning (CFC) paradigm, the behavioral response is analyzed following the association of a conditioned stimulus (CS) and an unconditioned stimulus (US). In particular, during the acquisition phase, an emotionally neutral CS stimulus is paired with a US (which can be, for example, a foot shock). In rodents, indicators of fear are typically assessed by a powerful conditioned fear behavior response expressed by a state of freezing, i.e., the absence of any movement except breathing. So, in conditioned rats, a single training session induces a freezing behavior response during the retrieval test, indicative of a fearful condition [21]. The animal learns and maintains the association between the new environment (SC) and the US for a long time.

The animals learn the connection between the CS and the US and obtain a fear response towards the environment. This process is called “contextual fear conditioning” (CFC) [21].

If, at this point, a signal (usually an auditory stimulus (CS)) is coupled to the US multiple times during training, the auditory stimulus (CS) will be separated from the environment and associated with the foot shock (US) to form the fear response to the auditory stimulus. This process is called “fear conditioning” [22,23]. Exposure to the CS alone is sufficient to initiate a fear response (CR). This is the retrieval phase of fear memory [24]. After retrieval, memory becomes destabilized, requiring new protein synthesis to recover, a process called “reconsolidation”. Reconsolidation has the function of stabilizing and integrating new information into long-term memory [25]. Moreover, a change in reconsolidation may represent an interesting therapeutic target for modulating the fear response associated with debilitating mental disorders. In particular, in this regard, subjects with PTSD are unable to weaken memories through reconsolidation [26].

Finally, once the animal acquires the fear response, repetition of the CS alone reduces the fear response: This is “extinction”, i.e., the establishment of a new competing memory, but not the elimination of the original fear memory.

Long-term potentiation (LTP) has also been suggested in fear-conditioned PTSD [27], as the blockade of NMDA receptors in the basolateral amygdala has been shown to prevent the acquisition of the fear-potentiated startle response [28]. Both long-term memory formation and conditioning cause changes in synaptic efficacy that are consequential to changes in neural transmission [29,30,31]. The memory of fear is regulated by a complex neurocircuitry, which unfolds along various brain areas including the hippocampus, amygdala, and prefrontal cortex [32,33,34,35,36]. The dorsal hippocampus and, more generally, the medio-temporal regions, are closely associated with the development and consolidation of memory [37,38]. Furthermore, the modulation of gene expression and epigenetic modifications in the hippocampus have been reported to be essential for the generation and consolidation of CFC memory traces [37,38]. Studies have suggested that the cerebellar areas are also involved. In particular, in the rodent model, it has been observed that the cerebellar structures are involved in the storage processes of conditioned responses [39]. The functional integrity of specific cerebellar regions is critical for the consolidation of fear conditioning [20,40,41].

## 4. Noradrenaline (NA)

Noradrenergic (NA) dysregulation has been shown to be present in PTSD. Individuals with PTSD have significantly higher norepinephrine levels than controls [42].

The use of a noradrenergic receptor blocker has been shown to be effective in reducing nightmares and reliving the symptoms of PTSD. It has also been shown that in PTSD, there are elevated cortisol levels, typically associated with a chronic stress condition, while lower cortisol levels immediately after trauma are predictive of the severity of PTSD [43]. This could be a stress control system based on a negative feedback regulatory mechanism. On the other hand, a reduction in cortisol levels in PTSD could lead to an increase in hypothalamic–pituitary–adrenal (HPA) axis activity, with an increase in catecholamines and a consolidation of trauma memories. This evidence is confirmed in animal models, where the administration of hydrocortisone immediately after exposure to stress factors results in a reduction in subsequent reactions similar to post-traumatic stress disorder. In agreement with this, there is evidence that females are twice as likely to develop PTSD than males [44]. Females show a greater noradrenergic response to aversive stimuli, a greater extent of context-enhanced fright, and greater amygdala reactivity to threatening stimuli. Furthermore, the menstrual phase influences PTSD phenomena, confirming that sex hormones play an important role in these conditions. Females with PTSD show impaired extinction learning in the mid-luteal phase (when progesterone and estradiol levels are elevated) [45]. One explanation as to why progesterone may facilitate emotional memories is that it binds to glucocorticoid receptors, thereby affecting the release of endogenous glucocorticoids.

Hence, catecholamines are influenced by sex steroid hormones [46,47]. In women, the HPA axis is more sensitized than in men, and women with PTSD had higher norepinephrine concentrations than controls. This may be because estrogens may increase NA in target regions of the locus coeruleus by improving norepinephrine synthesis capacity while decreasing NA degradation, potentially increasing arousal in women [48].

Similarly, NA affects the acquisition of CFC, and this is supported by evidence that neurons in the *locus coeruleus* show increased expression of *Fos* following CFC [49]. Furthermore, lesions of the *locus coeruleus* modify contextual fear conditioning [50]. Genetic and pharmacological studies related to NA have confirmed that it regulates contextual fear learning. The *locus coeruleus*-NA system would therefore seem crucial for the simultaneous acquisition, consolidation, and extinction of the memory of fear. This concept is likewise supported by the high expression of adrenergic receptors in the hippocampus that encode contextual information and by the ability of NA to modulate LTP [51,52].

## 5. Dopamine

The region of the brain that constitutes the fulcrum of fear conditioning, where the memory trace is formed, and which underlies emotional and associative learning is the amygdala. Evidence has suggested that dopaminergic pathways are involved in the aversive learning behavioral paradigm in rodent study models [53,54,55,56]. Through the use of positron emission tomography and functional magnetic resonance imaging, Frick et al. showed [57] dopamine release in the amygdala and striatum in humans during fear learning. This is in line with the observation of Uhlén et al. [58] of increased expression of dopamine 2 receptor in the basolateral nucleus of the amygdala. On the other hand, low dopaminergic function has been associated with a higher risk of PTSD [59,60]. In human subjects with Parkinson’s and therefore with a reduction in dopaminergic transmission, fear processing is impaired and recovers after dopaminergic treatment [61]. In addition, it is known that 100 times more dopamine is released during combat stress than at rest. The functional significance of dopamine may be to facilitate long-term potentiation and conditioning by enhancing the formation of both fear memory and nonassociative learning [62,63].

## 6. Gamma-Aminobutyric Acid (GABA)

Many studies have been aimed at analyzing the role of GABA in fear memory. Long-term retention of fear memories correlates with impaired GABAergic neurotransmission. Although sometimes conflicting, findings have suggested that the timing of the application of GABA and/or GABAergic drugs, their dosage, and the brain region are crucial determinants of GABA effects [64].

The types of GABA receptors have been identified, namely, GABA-A, GABA-B, and GABA-C. The GABA-A and GABA-C receptors are ligand-linked ion channels, while the GABA-B receptor is of the metabotropic type, coupled to a G protein [65]. In particular, the GABA-A receptor plays a role in the amygdala and prefrontal cortex where it mediates fear memory.

The administration of GABAergic drugs before or immediately after extinction training blocks response inhibition, suggesting that GABA may disrupt the acquisition and consolidation of extinction memories, and also suggesting that there is interference with extinction memory consolidation processes. However, other studies have observed conflicting results, possibly because they used high doses of GABA [66] or infused it into brain regions that may have inhibited fear expression [67]. Conversely, when GABAergic drugs are administered prior to testing, the conditioned response is less than that of vehicle infusion, suggesting facilitation of the response inhibition cycle. This happens because they reduce the anxiety condition, facilitating recovery or possibly facilitating the inhibition of the fear response. When GABAergic transmission is downregulated prior to testing, the fear response increases, suggesting interference with the response inhibition mechanism [68]. Thus, the processes that mediate memory persistence after initial fear learning, memory reactivation, and extinction training all depend on impaired GABAergic transmission. The downregulation of GABAergic transmission likely occurs in the amygdala, hippocampus, or prefrontal cortex. Finally, it has also been suggested that GABA downregulation may modulate memory storage by facilitating NE release [64].

On the other hand, the GABAergic system is known to play a role in depression, anxiety, and other mental disorders. Recent studies reported that reduced levels of GABA are associated with stress responses [69].

## 7. Glutamate

Studies have reported alterations in glutamatergic neurotransmission associated with dysfunction of the brain networks underlying PTSD memory and emotional processes [70,71]. It has been shown that traumatic situations such as chronic stress tend to decrease the density of glutamatergic synapses, as well as being associated with a decrease in gray matter, in particular, at the level of the prefrontal cortex [72,73].

On the other hand, under stress conditions, there are glutamatergic modifications that can lead to deficits in associative learning and memory [74,75]. This has led to the consideration that markers of metabolism and excitotoxicity produced by glutamate may be of particular relevance to memory-related symptoms in PTSD.

Synaptic changes have been identified in temporal regions in the amygdala area. The blockade of NMDA receptors in the basolateral amygdala has been shown to prevent the acquisition of the initial fear response [76].

Although there is evidence for the regulation of metabotropic glutamatergic receptors of both hippocampal plasticity and conditioned fear behavior, in reality, the relationship between these two phenomena has not yet been fully elucidated.

## 8. Acetylcholine

The role of neurotransmitter acetylcholine (ACh) in learning and memory phenomena is well known. In particular, in this context, memory reconsolidation is strengthened following an enhancement of the cholinergic system [77]. Evidence has suggested the role of muscarinic cholinergic receptors at the level of brain areas involved in the regulation of CFC formation, i.e., in the amygdala, hippocampus, and prefrontal cortex. It has been observed that the functionality of the M1 muscarinic receptors is required for the contextual extinction of fear [78]. It has also been found that pharmacological treatment with a muscarinic cholinergic receptor antagonist, such as atropine, impairs the retention of older fear memory in animal models of CFC [79]. On the other hand, the role of nicotinic cholinergic receptors in fear learning and extinction remains more uncertain [80].

## 9. Serotonin

Experimental evidence also supports the involvement of serotonin (5-hydroxytryptamine, 5-HT) learning processes in fear responses and anxiety [9].

Mice with defects in the development of the 5-HT system have been observed to exhibit anxiety-like behaviors and fear memory [81,82,83]. This has led to the hypothesis that the 5-HT system plays a crucial role in the regulation of fear memory in rodents [36]. From an anatomical point of view, a direct connection has been highlighted between the cerebral sources of serotonin, nuclei of the dorsal raphe and brainstem and medial prefrontal cortex, the hippocampus, and the amygdala, which control responses to anxiety and fear [84,85]. This evidence could suggest that the exaggerated context-dependent fear memory and shock reactivity resulting from reduced 5-HT could be based on an alteration of the raphe-hippocampal innervation.

## 10. Glycine

Glycinergic neurotransmission may also play an important role in conditioned fear [86]. The effects of selective glycine transporter-1 inhibitors on contextual fear conditioning were studied [87]. In particular, in the hippocampus, the levels of glycine transporter 1 (Gly-T1) and vesicle-associated membrane protein 2 (VAMP2) mRNA in rats subjected to prolonged stress as a model of PTSD are significantly increased compared to control rats [88].

## 11. Histamine

Of the four known histaminergic receptors, H1, H2, and H3 have been identified in the brain. In particular, H1 and H2 receptors potentiate excitatory responses while H3 receptors mediate inhibitory actions [89]. Interestingly, histamine modulates hippocampal activity [90]. Various studies have highlighted the role of histamine in regulating the consolidation of emotional memories. For example, rats injected with histaminergic H3 receptor antagonists at the basolateral amygdala, immediately after being subjected to contextual fear conditioning, showed a significant alteration in memory consolidation, while histamine infusion into the hippocampus or basolateral amygdala restores long-term memory [91,92]. A recent study combined experimental tools with mathematical models and revealed that an increase in serotonin concomitant with a reduction in histamine constitutes the best chemical strategy to restore serotonin to pre-stress levels in an animal stress model [93]. Therefore, such a study suggested and supported a co-modulatory relationship between serotoninergic and histaminergic neurotransmission under conditions of chronic stress in mice.

## 12. Purines

Last but not least, the purinergic system also has a significant role in the CFC model. This neurotransmission system is made up of two large families of receptors: P2X and P2Y activated by adenosine triphosphate (ATP). These receptors are distributed in many brain areas, including those involved in stress-related emotions and behaviors and, therefore, in conditions of stress and fear. Currently, there are few studies that have investigated the purinergic system in CFC. In particular, a recent study has shown that pharmacological or genetic blockade of the purinergic receptor P2X7R promotes anxiety-provoking states, in combination with alterations in extinction learning. The study therefore suggested the possibility of treating stress-related psychiatric disorders [94].

It has also been observed that stressful conditions can activate P2X7 purinergic receptors on microglia to induce inflammatory states as well as behavioral modifications [95].

**Figure 1:**
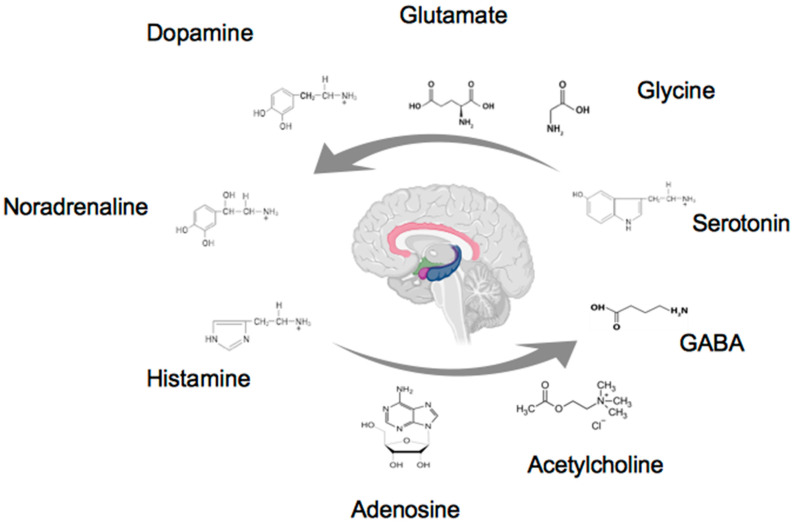
The figure reproduces the human brain, and the areas of the limbic system involved in CFC and PTSD are colored. The classical neurotransmitters discussed in the manuscript are reported. The arrows are intended to highlight the integration and coordination of neurotransmitters in stress and fear responses (created by BioRender).

In line with this evidence, P2X7R mRNA expression was detected in the blood of untreated subjects with treatment-resistant major depression [96] (Figure 1 and Table 1).

## 13. Signaling Pathways Associated with PTSD

In this context, it is important to report the decisive role of the stress hormone cortisol and the dysregulation of the hypothalamic–pituitary–adrenal (HPA) axis, the stress system whose activation is fundamental for the survival of an individual in the context of internal or external threats that affect homeostasis [97]. Cortisol modulates emotional learning and memory processes, improving the consolidation of declarative memories [98]. Cortisol administration reduces fear memory, and its reduction can hinder the extinction of fear responses associated with traumatic memories [43,99].

The crucial brain region involved appears to be the amygdala [100]. On the other hand, excitatory input from the anterior cingulate cortex and inhibitory input from the ventromedial prefrontal cortex modulate the expression of fear and extinction memories via the amygdala [101,102]. This circuit receives contextual information from the hippocampus [103,104]. Generally, lower cortisol levels were found in subjects suffering from PTSD compared to unexposed controls.

Specific molecular targets for potential therapeutic modulation are related to cellular networks, especially signaling pathways that mediate responses to trauma and severe stress with their potential contribution to the etiology of PTSD. This area has been reviewed by Hauger et al. [105], and more recently, by Girgenti et al. [106].

**Figure 2:**
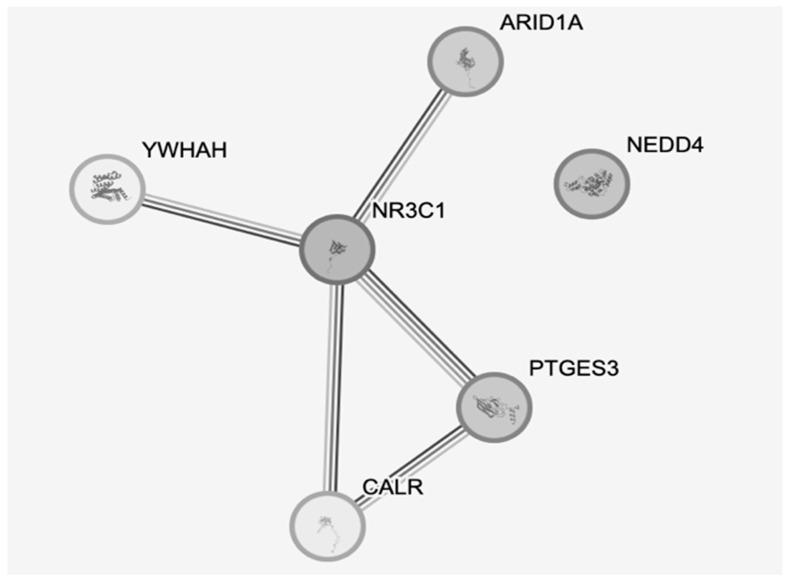
Schematic representation of the GR pathway (obtained using STRING-db.org, accessed 30 September 2023). YWHAH, Tyrosine 3-Monooxygenase/Tryptophan 5-Monooxygenase Activation Protein Eta; ARID1A, AT-rich interaction domain 1A; NR3C1, nuclear receptor subfamily 3 group C member 1; PTGES3, Prostaglandin E synthase 3; CALR, calreticulin.

In particular, it has been highlighted that sensitization of glucocorticoid receptor (GR) signaling and impairment of a multiplicity of GR modulators, such as proteins encoded by FKBP5, STAT5B, Bcl-2, and Bax genes, have been involved in PTSD pathophysiology [107]. In addition, Akt, NFκB, MKP-1, and p11, which are G protein-coupled receptor (GPCR) pathway molecules, can favor or anticipate sustained high-anxiety conditions and depressive-like responses following severe stress [105]. This network is schematically illustrated in Figure 2 with the key proteins involved in signaling. Importantly, glucocorticoids are primary stress hormones that regulate numerous physiological processes aimed at maintaining homeostasis. Both the physiological and pharmacological actions of glucocorticoids are mediated by GR, which is a member of the nuclear receptor superfamily of ligand-dependent transcription factors. There are numerous synthetic derivatives of these hormones that have been used in the clinic for treating inflammatory diseases, autoimmune disorders, and hematological cancers. Therefore, some of the agents, with proper care, may be repurposed for the management of PTSD [100].

**Figure 3:**
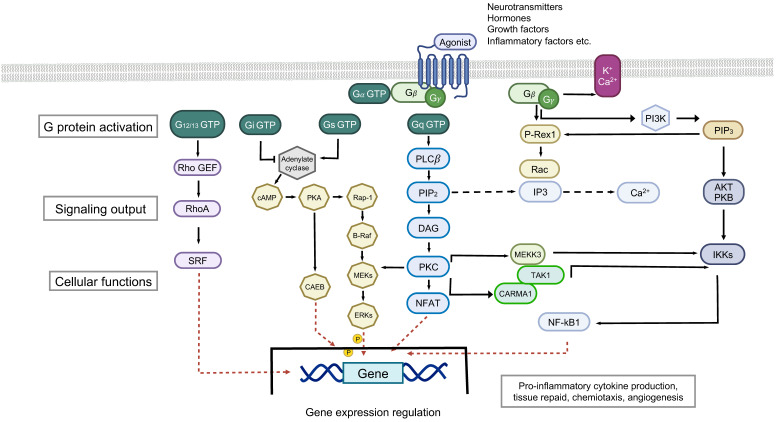
Schematic representation of the GPCR pathway. PIP3, phosphatidylinositol (3,4,5)-trisphosphate; RAC, a subfamily of the Rho family of GTPases; P-REX, Phosphatidylinositol-3,4,5-Trisphosphate Dependent Rac Exchange Factor; IP3, inositol 1,4,5-trisphosphate; AKT PKB, Ser/Thr kinase *AKT*, also known as protein kinase B (*PKB*); IKK, IκB kinase; RHO CEF, Rho GTPase chicken embryo fibroblasts; SRF, Serum Response Factor; cAMP, Cyclic adenosine monophosphate; PKA, *protein* kinase A; CAEB, Coxiella burnetii effector *protein;* B-RAF, signal transduction serine/threonine-specific protein kinase; RAP-1, Ras-proximate-1 or Ras-related protein 1; MEK, mitogen-activated protein; ERKs, extracellular signal-regulated kinases; PLCb, Phospholipase C Beta; PIP2, phosphatidylinositol 4,5-bisphosphate; NFAT, nuclear factor of activated T cells (NFAT) protein; NfkB, Nuclear factor kappa-light-chain-enhancer of activated B cells; CARMA1, Caspase recruitment domain-containing protein 11; TAK, Transforming growth factor-β (TGF-β)-activated kinase 1 (created by BioRender).

The GPCR pathway is illustrated in Figure 3. Notably, GPCRs form the largest and most diverse group of membrane receptors not only in humans but also in all eukaryotes. For this reason, comprehensive elaboration of their structures and forms is outside this PTSD-focused review. Suffice it to say that these cell surface receptors act as receivers of signals in the form of electromagnetic energy, peptides, lipids, sugars, and proteins. These signals instruct the receiving cells regarding the presence or absence of essential nutrients or energy sources in their environment. GPCRs play significant roles in numerous physiological functions in the human body. It is, therefore, not surprising that an estimated one-third or more of all approved pharmacological agents involve binding to GPCRs. GPCRs share a common structural design that has been conserved over the course of evolution. In the human body, nearly 1000 different GPCRs are expressed, and each one of them is highly specialized for the efficient detection of a particular signal.

## 14. Treatments for PTSD

The use of psychological interventions is generally considered to be a first-line approach to the treatment of PTSD [108]. While various psychotherapies are available for PTSD patients, cognitive behavioral therapy is currently accepted as having the strongest clinical evidence for reducing the symptoms of PTSD and has been shown to be more effective than any non-pharmaceutical treatment. In terms of pharmacotherapeutic approaches to PTSD, antidepressants have been the medications of choice, with the strongest empirical evidence available for the use of selective serotonin reuptake inhibitors (SSRIs). Specific approval for the treatment of PTSD by the Food and Drug Administration (FDA) has only been given to two drugs so far, namely sertraline and paroxetine [109]. All other medications are prescribed by doctors to PTSD patients as off-label and only have empirical support. These off-label medications in use include the SSRI fluoxetine and the serotonin norepinephrine reuptake inhibitor venlafaxine, which are recommended as first-line treatments. Venlafaxine acts primarily as an SSRI at lower dosages and as a combined SNRI at higher dosages. Although SSRIs are associated with an overall response rate of approximately 60% in PTSD patients, only between 20% and 30% of patients experience complete remission [110]. In patients with PTSD who were treated with extended-release (ER) venlafaxine, the response rate reached was as high as 78%, with a remission rate of 40% [111].

## 15. Summary and Conclusions

This review focused on fear conditioning, which constitutes a well-developed and consolidated paradigm for the study of the neurobiological mechanisms underlying emotions and, in particular, a form of learning through which a dangerous condition is recognized. On the other hand, studies on the synaptic plasticity phenomena underlying fear conditioning present neural circuits underlying this learning model that are strongly correlated with pathological fears, anxiety states, and PTSD. In both humans and the rodent model, fear conditioning and context rely on dependent neurocircuitry in the amygdala and prefrontal cortex, cingulate gyrus, and hippocampus. This review provides a collection of information on the role played by classical neurotransmitters in the contextual conditioning model of fear and, therefore, in PTSD. It appears that the neurochemical basis of PTSD includes the altered regulation of catecholamines, serotonin, amino and purine neurotransmitters, and the HPA axis, and each of these resides in brain networks that integrate and coordinate responses to stress and fear.

The review has also provided a brief overview of both behavioral and pharmaceutical treatments currently available for PTSD.

From this overview, it emerges that the biological alterations that affect patients suffering from PSTD are many and that these are likely structurally and functionally connected to each other.

Future studies will shed further light on the cellular and molecular mechanisms underlying this disorder, including by continuing to exploit the animal conditioning paradigm, with the aim of finding a definitive cure for patients with PTSD, which represents a significant health and social burden due to its severity, chronicity, and comorbidity with conditions such as major depression.

## Figures and Tables

**Table 1:** Neurotransmission and neuroendocrine axis in CFC/PTSD.

Neurotransmitter	Role in CFC/PTSD	Refs.
Acetylcholine	Regulates CFC formation Increases under stress	[77,78,79,80]
Dopamine	Contributes to fear learning Increases in stress condition	[53,54,55,56,57,58,59,60,61,62,63]
GABA	Destroys consolidation of memories Reduced under stress	[64,65,66,67,68,69]
Glutamate	Blocking its transmission prevents acquisition of reduced fear	[70,71,72,73,74,75,76]
Glycine	Its reduction increases PTSD	[86,87,88]
Histamine	Consolidates memory Contributes to stressful condition	[89,90,91,92,93]
Noradrenaline	Increases CFC Increases in stressful condition	[42,43,44,45,46,47,48,49,50,51,52]
Purine	Controls anxiety and fear	[94,95,96]
Serotonin	Controls responses to anxiety and fear	[9,81,82,83,84,85]
Hypothalamic-pituitary-adrenal axis (HPA)	Drives fear processing Regulates response to stress	[44,45,46,47,48,97,98,99]
